# A call to expand the dimensions of halide-philicity

**DOI:** 10.1128/mbio.01084-26

**Published:** 2026-06-17

**Authors:** Lawrence P. Wackett, Randy B. Stockbridge

**Affiliations:** 1 Department of Biochemistry, Biophysics & Molecular Biology, University of Minnesota 5635, Minneapolis, Minnesota, USA; 2 Department of Molecular, Cellular, and Developmental Biology, University of Michigan 1259, Ann Arbor, Michigan, USA; Georgia Institute of Technology, Atlanta, Georgia, USA

**Keywords:** halophile, halide, organohalide, microorganisms, bacteria, salt, stress

## Abstract

Microbiology research has historically associated the term halophily with sodium chloride. Today, other halides are taking on increasing importance with the broader use of other halide anions, their oxyanions, and thousands of organohalides. This has led to a wider and human-altered distribution of halides and organohalides in the environment, as well as increased efforts to bioengineer microbes to synthesize or degrade organohalides and to tolerate their associated halide salts. In this context, it needs to be more widely appreciated that microbial responses to fluorine, chlorine, bromine, or iodine are vastly different. Moreover, the way microbes handle a given halide may differ when they are paired with different counter cations. In addition, the microbial responses may differ when the halogens are in the external environment or liberated inside the cell by cytoplasmic enzymes, for example, by dehalogenases. In this commentary, we seek to highlight these differences and offer a call for future research.

## COMMENTARY

Science is often perceived as ahistorical, but as a human endeavor, scientific knowledge proceeds by historical contingency (1). In this context, we believe it is important to expand the concept of halophilicity in reference to microbial responses to halogens. The need is spurred by the diverse and accelerating uses of fluorine, chlorine, bromine, and iodine by human society. Recent research has revealed substantially varied responses of microbes to the inorganic and organic forms of the different halogens. Below, we discuss historical developments that naturally led to the term “halophilicity” and the imperative to appreciate and expand the dimensions of halide-philicity.

Chlorine was first identified from mineral acid, iodine from kelp, and bromine from brine (2). Although the element fluorine was not isolated until 1886, the Swedish chemist Berzelius started using the term halogens for these elements and halides for their anionic forms in 1826. This nomenclature has persisted until today. By the early 1900s, microbiologists were using the term “halophile” to describe bacteria isolated from briny environments (3). Early papers, and the vast majority of studies on halophiles since, have focused on one halide, chloride, with sodium chloride as the main growth medium reagent for selective bacterial isolations (4). Today, microbial adaptations to other halides have taken on increased importance with broad use of all the halides in industry, biotechnology, and pharmacology (5), especially given the wide distribution of halides and organohalides in human-altered environments. Although numerous studies have conflated NaCl tolerance with the ability to withstand other halides, we argue that a more nuanced view of the vastly different microbial responses to fluoride, chloride, bromide, or iodide and their counterions is warranted.

Microbes encounter a diversity of halides in Earth’s environments (Table 1). Fluoride is the most common halide in the Earth’s crust, and though it is generally found at low levels in the oceans, it reaches concentrations as high as 100 mM in the lakes of the Rift Valley due to extensive volcanic inputs (6). A few microbes use fluoride to synthesize mono-fluorinated toxins, such as fluoroacetate (7), and these organisms have evolved mechanisms to evade fluoride toxicity, such as the temporal separation of cellular metabolism from organofluorine biosynthesis (8). Chloride is the most common halide in seawater and many natural brines. In addition to chloride anion, chlorine is also found in biological systems as perchlorate, hypochlorite, and thousands of chlorinated natural products (Table 1) (9). Bromide is uncommon in the Earth’s crust but common in briny lakes and the oceans. In the Dead Sea, chloride is the major anion, but bromide levels up to 75 mM have been identified (5). Numerous organobromine compounds are produced by marine organisms. Iodine cycles biologically between iodide, iodine, triiodine anion, iodate, and organoiodine compounds, and most forms of iodine exist in the global oceans (10).

**TABLE 1:** Relevance of inorganic and organic halides to human society and microbial physiology (11)^*a*^

Halide	Relevant inorganic form	Tolerance limit (external)	Tolerance limit (internal)	No. of natural products	Uses in society	Annual usage (kg)
F^−^	F^−^	0.2 M	100 μM	10^1^	Electronics, batteries, pharmaceuticals, pesticides, >22 million fluorinated organics	8 billion
Cl^−^	Cl^−^ ClO^−^ ClO_4_^−^	5 M	4.5 M	10^4^	Chemical production, road deicing, food processing	270 billion
Br^−^	Br^−^ BrO^−^	4 M	Unknown	10^3^	Computers, telephones, insulation, agricultural chemicals, batteries	450,000
I^−^	I^−^ I_2_^−^ I_3_^−^ IO_3_^−^	Depends on ionic form	Depends on ionic form	10^2^	X-ray imaging, LCD screens, supplements	41,000

^*a*^ Tolerance (12–15) and utilization of halides for natural product biosynthesis (11) shows a very large range for fluoride, chloride, bromide, and iodide.

Additional environmental factors, including pH, counterion, and the abundance of divalent metals like calcium or magnesium, differentially impact microbial tolerance to each of these halides. For example, some microbes that are highly resistant to NaCl exhibit even greater sensitivity to MgCl_2_ than standard lab strains (16), and fluoride toxicity increases together with the acidity of the medium (17). Moreover, the microbial response to external halides is quite different than the response to halides liberated inside the cell by cytoplasmic enzymes like dehalogenases, an issue that has become especially pertinent in modern times with the extensive production of organohalide compounds for industrial purposes (Table 1). The major halogens in this context are chlorine in pesticides and fluorine in pesticides, drugs, and other materials, including the so-called per- and polyfluorinated alkyl substances (PFAS). First introduced commercially in the late 1940s, PFAS became widely used in pesticides, pharmaceuticals, and common consumer products (18). The biodegradation of these compounds liberates halide salts. While chloride liberation is not toxic, the liberation of fluoride anion disrupts cellular metabolism by binding tightly to essential enzymes that coordinate magnesium and calcium. Fluoride is produced during enzymatic C-F bond cleavage, and this release of a toxic halide anion is likely to limit the biodegradation of highly fluorinated compounds, such as PFAS (12). Thus, the microbial response to external halides is quite different than the response to halides liberated inside the cell by cytoplasmic enzymes like dehalogenases (19).

For these reasons, we propose that “halide-philicity” be understood as a multi-dimensional property that depends on the specific anion, its environmental counterions, and its mechanism of exposure. Some microbes have evolved very high tolerance to chloride, and it has been proposed that chloride tolerance is bounded only by the solubility limit of chloride in brines (13). While many microbes withstand high osmotic pressure by synthesizing or importing osmolytes, such as glycine betaine, betaine, and choline, other microorganisms achieve osmotic balance by active import of halide salts by proteins, such as the halorhodopsins, which are permeable to both chloride and bromide (20). Bacteria that accumulate these halides must be able to withstand high internal ionic strength, reaching up to 4 M, which requires an entirely different set of adaptations. Halides, along with the common counterion potassium, are typically chaotropic, causing protein denaturation. Bacteria that import salts often exhibit a genome-wide enrichment of acidic amino acids to compensate for the destabilizing effect of high salt (21), although the underlying physical mechanisms for this stabilizing effect remain unclear (22). Bromide and iodide are increasingly chaotropic compared to chloride and, thus, select for microorganisms that can better withstand chaotropic stress (14). In addition, organic or inorganic cations, such as glycine betaine or sodium, can serve as compensating kosmotropes (23) that increase protein stability and mitigate the effects of chaotropic ions like chloride, bromide, or iodide.

Fluoride tolerance represents an independent dimension of halide-philicity and is mainly conferred by fluoride exporters that maintain low internal fluoride (24). However, many other metabolic and homeostatic adaptations have been identified that help microbes withstand and even assimilate fluoride ion (12). An organism’s fluoride tolerance is tightly linked to the environmental counterions and pH. For example, some halophiles isolated from Lake Magadi in the Rift Valley, which has the highest known fluoride levels on Earth, do not possess genes that encode fluoride exporters—the main counterions present, magnesium and calcium, form stable complexes with fluoride that render the fluoride mostly nontoxic. In contrast, fluoride becomes increasingly permeable to the cell membrane under acidic conditions, decreasing the microbial limit of tolerance by orders of magnitude at low pH (17). Finally, halide-philicity can be understood in terms of microbe’s ability to utilize halides or their oxyanion derivatives (Table 1). Some microbes consume fluoride, chloride, or bromide to synthesize organohalide natural products that they use in a plethora of physiological contexts (Table 1) (25). Other microbes use oxyhalides as electron acceptors for anaerobic respiration and possess highly selective enzymes, including perchlorate reductase, chlorite dismutase, or iodate reductase (15, 26). For such organisms, halides serve not only as physiological stressors, but also as energy sources, highlighting the complex selective pressures in such niches.

These properties all reflect independent determinants of fitness in halide-rich environments. The interplay of the salts present, including their specific salt chemistries, means that each unique brine exerts different, non-cumulative selective pressures, and microbes isolated from different salty niches have inherently different tolerances to different halides. While a broader genetic understanding of halide tolerance mechanisms has been built over many years, more work is needed to understand how microbes integrate these responses in physiological and biotechnological contexts. In particular, gaps remain in characterizing molecular responses to bromide and iodide, how microbes respond to admixtures of halide ions that mimic real-world conditions, and the influence of counterions on resistance. In this context, we advocate here for a shift to a multidimensional view of halophily to consider the individual effects of halides, their forms and their concentrations, and the mechanisms of microbial exposure.
